# Dysfunction of parvalbumin-expressing cells in the thalamic reticular nucleus induces cortical spike-and-wave discharges and an unconscious state

**DOI:** 10.1093/braincomms/fcac010

**Published:** 2022-01-28

**Authors:** Manal S. Abdelaal, Mitsuharu Midorikawa, Toru Suzuki, Kenta Kobayashi, Norio Takata, Mariko Miyata, Masaru Mimura, Kenji F. Tanaka

**Affiliations:** 1 Department of Neuropsychiatry, Keio University School of Medicine, Tokyo 160-8582, Japan; 2 Division of Neurophysiology, Department of Physiology, School of Medicine, Tokyo Women’s Medical University, Tokyo 162-8666, Japan; 3 Division of Brain Sciences, Institute for Advanced Medical Research, Keio University School of Medicine, Tokyo 160-8582, Japan; 4 Section of Viral Vector Development, National Institute for Physiological Sciences, Okazaki 444-8585, Japan

**Keywords:** animal model of absence seizure, tetracycline-controllable gene induction, inhibitory opsin, T-type calcium channel, rebound burst firing

## Abstract

Spike-and-wave discharges and an accompanying loss of consciousness are hallmarks of absence seizure, which is a childhood generalized epilepsy disorder. In absence seizure, dysfunction of the cortico-thalamo-cortico circuitry is thought to engage in abnormal cortical rhythms. Previous studies demonstrated that the thalamic reticular nucleus has a critical role in the formation of normal cortical rhythms; however, whether thalamic reticular nucleus dysfunction leads directly to abnormal rhythms, such as epilepsy, is largely unknown. We found that expressing the inhibitory opsin, archaerhodopsin, including in the thalamic reticular nucleus, caused abnormal cortical rhythms in *Pvalb*-tetracycline transactivator::tetO-ArchT (PV-ArchT) double transgenic mice. We validated the PV-ArchT line as a new mouse model of absence seizure through physiological and pharmacological analyses, as well as through examining their behavioural features. We then discovered that archaerhodopsin expression exclusively in thalamic reticular nucleus parvalbumin-positive neurons was sufficient to induce cortical spike-and-wave discharges using adeno-associated virus-mediated thalamic reticular nucleus targeting. Furthermore, we found that archaerhodopsin expression impaired rebound burst firing and T-current in thalamic reticular nucleus parvalbumin-positive cells by slice physiology. Although T-current in the thalamic reticular nucleus was impaired, the T-current blocker ethosuximide still had a therapeutic effect in PV-ArchT mice, suggesting a gain of function of T-type calcium channels in this absence seizure model. However, we did not find any over- or misexpression of T-type calcium channel genes in the thalamus or the cortex. Thus, we demonstrated that thalamic reticular nucleus dysfunction led to an absence seizure-like phenotype in mice. In a final set of experiments, we showed that the archaerhodopsin-mediated absence seizure-like phenotype disappeared after the removal of archaerhodopsin by using a time-controllable transgenic system. These data may provide a hint as to why many absence seizures naturally regress.

## Teaser

Abdelaal *et al*. demonstrated that the loss of function of parvalbumin-expressing neurons in the thalamic reticular nucleus is sufficient to induce an absence seizure-like phenotype in mice. The time-controllable, loss of function reveals a gating of disease progression and regression in the absence seizure model.

## Graphical Abstract

**Graphical abstract:**
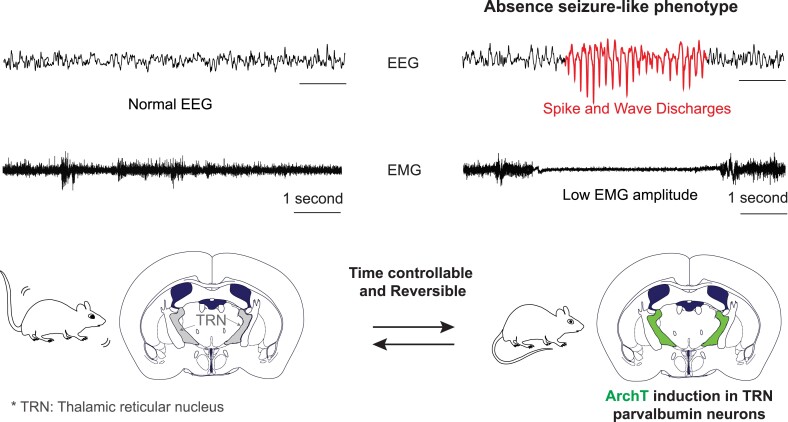

## Introduction

Absence seizure is a childhood-onset neurological disorder and is classified as a generalized non-motor seizure according to the International League Against Epilepsy Classification of 2017.^1^ The loss of consciousness for a few seconds is a clinical feature of absence seizures. This is associated with spike-and-wave discharges (SWDs), which are the physiological hallmark of absence seizures.^2–4^

Cortico-thalamo-cortical networks are involved in the pathophysiology of SWDs.^5,6^ In rodents, these networks include two types of excitatory neurons, corticothalamic (CT) and thalamocortical (TC) neurons, and one type of inhibitory neuron, thalamic reticular nucleus (TRN) neurons.^7,8^ TRN inhibitory neurons receive excitatory inputs from both CT and TC neurons, but send axons only to TC neurons. TRN neuronal activities are divided into two modes, tonic and burst firing. Burst firing is generated by T-type Ca^2+^ channels, and T-type Ca^2+^ channel-dependent burst firing is involved in the initiation of cortical sleep spindles through the rhythmic inhibition of TC neurons.^9^ In addition to its role on sleep homeostasis, burst firing of the TRN is thought to associate with cognition, attention and consciousness, and dysregulation of the TRN may cause neuropsychiatric diseases including absence seizures.^10^

The aetiology of absence seizure is not clear, and there are several theories for the generation of SWDs. Although the role of the TRN in cortical rhythm generation has been widely accepted from the viewpoint of sleep spindle generation, it is not known whether TRN dysfunction *per se* leads to SWDs in absence seizures. Here, we sought to demonstrate that dysfunction in the TRN alone is capable of generating absence seizures. To this end, we exploited a cell type-specific toxic gene induction system in mice. We found that an inhibitory opsin, ArchT (archaerhodopsin from Halorubrum strain TP009), rendered TRN parvalbumin (PV)-positive cells abnormal and we used ArchT as a toxic agent in this study. Moreover, we demonstrated that mice expressing ArchT in the TRN can serve as a new animal model of absence seizure.

## Materials and methods

All animal procedures were conducted by the National Institutes of Health Guide for the Care and Use of Laboratory Animals and approved by the Animal Research Committee of Keio University School of Medicine. We used male and female mice between 2 and 8 months of age. Furthermore, mice were kept on a 12 /12 h light/dark cycle in their home cage.

### Transgenic animals

We generated *Pvalb*-tTA::tetO-ArchT (hereafter called PV-ArchT) double transgenic mice by crossing *Pvalb*-tTA mice^11^ with tetO-ArchT-EGFP mice.^12^ PV-ArchT mice were generated to express ArchT, an inhibitory opsin, specifically in PV neurons. For the control in the slice physiology experiment, we used *Pvalb*-tTA::tetO-yellow cameleon (YC)^13^ double transgenic mice. *Pvalb*-Cre mice^14^ were used for AAV-mediated ArchT expression. Genotyping methods were previously described.^11–14^

### Immunohistochemistry

All mice were deeply anaesthetized and then perfused with 4% paraformaldehyde in a phosphate-buffered solution (PBS). Brains were removed from the skull, post-fixed overnight in the same fixative and cryoprotected in 20% sucrose/PBS overnight. Brains were then frozen and cut at 25 µm thickness on a cryostat. Sections were mounted on silane-coated glass slides (Matsunami Glass, Japan). For double immunohistochemistry (IHC), sections were incubated overnight at room temperature in a mixture of primary antibodies: anti-green fluorescent protein (GFP) antibody (goat polyclonal, 1:250, Rockland Immunochemicals Inc., USA) and anti-PV antibody (mouse monoclonal clone 235, 1:1000, Swant Inc., Switzerland). On the second day, sections were incubated for 2 h at room temperature in a mixture of secondary antibodies: donkey anti-goat IgG-Alexa 488 antibody (1:1000, Invitrogen, USA) and donkey anti-mouse IgG-Alexa555 antibody (1:1000, Invitrogen). Fluorescent images were obtained with a confocal microscope (FV3000, Olympus) or an All-in-one Fluorescence Microscope (BZ-X710, Keyence).

### EEG and EMG electrode implantation and recording

Mice were anaesthetized with a mixture of ketamine and xylazine (100 and 10 mg/kg, respectively, intraperitoneally), and then fixed on a stereotaxic apparatus (Narishige Scientific Instrument Lab, Japan). For EEG electrode implantation, craniotomies were prepared ∼1 mm in diameter for signal and reference electrodes. Positions of craniotomies were as follows: cortical signal electrodes were located above the somatosensory area [anteroposterior (AP) −1.5 mm, mediolateral (ML) ± 2.0 mm from Bregma] and reference electrodes were positioned at (AP −6.1 mm, ML ± 0.5 mm from Bregma). Cortical EEG electrodes were placed above the dura mater. For local field potential (LFP) electrode implantation in the thalamus, tungsten wires were inserted at AP −0.82 mm, ML ± 0.9 mm and dorsoventral (DV) 3.2 mm from bregma. EMG electrodes were inserted into trapezius muscles. Electrodes were fixed with dental acrylic (Super-Bond C&B Sun Medical, Shiga, Japan) and dental cement (UNIFAST II, GC, Japan). After fixation, all electrodes were protected with dental silicone. Mice were allowed to recover for 1 week before measurements were taken.

EEG/EMG recordings were performed in freely moving mice in their home cage for 2 h/day. EEG and EMG signals were amplified 500 times, band-pass filtered (1–3000 Hz for EEG; 1–1000 Hz for EMG) (Model 3000, A-M systems; DAM50, World Precision Instruments, USA). Analysis was performed using the in-house software written in MATLAB (2018b; MathWorks).

### Pharmacological intervention

Ethosuximide (Tokyo Chemical Industry Co., Japan) was dissolved in normal saline and injected intraperitoneally to mice at 100 mg/kg. Baclofen (Sigma-Aldrich Japan, Japan) was dissolved in normal saline and injected intraperitoneally to mice at 20 mg/kg.

### Adeno-associated virus production and purification

Adeno-associated virus (AAV) vectors were produced using the AAV Helper-Free System (Agilent Technologies, Inc., USA) and the detailed methods of AAV production and purification were previously described.^15^ The pAAV-CAG-DIO-ArchT-GFP plasmid was purchased from Addgene (ID: 28307; USA). The titre of AAVDJ-CAG-ArchT-GFP was 1.1 × 10^13^ vg/ml.

### Cre-mediated ArchT expression

Methods for anaesthesia and stereotaxic surgery were the same as for implanting EEG and EMG electrodes. A glass capillary containing the virus solution (AAVDJ-CAG-DIO-ArchT-GFP) was inserted bilaterally into the TRN (AP −0.8, ML ± 1.6 and DV 3.6 mm) from the brain surface of *Pvalb*-Cre mice and the 150 nl solution was injected by the Nanoliter2010 Microinjection Pump (WPI, USA). The capillary was held in place for 20 min after injection to decrease the spreading of the virus.

### Patch clamp of brain slices

For control experiments, we generated *Pvalb*-tTA::tetO-YC double transgenic mice (PV-YC). Acute brain slices were prepared from either PV-ArchT or PV-YC juvenile mice. Fresh-prepared brain slices from postnatal day P21 to P26 male mice were superfused with oxygenated artificial cerebrospinal fluid (in mM: 125 NaCl, 26 NaHCO_3_, 20 glucose, 2.5 KCl, 1.25 NaH_2_PO_4_, 1.2 MgC1_2_, 2CaCl_2_) and recorded between the temperatures 30 and 32°C. Cells were whole-cell current-clamped or voltage-clamped using an EPC10/2 amplifier (HEKA, Germany), controlled by the PatchMaster software (HEKA, Germany). The patch pipettes were filled with intracellular solution containing (in mM): 150 K-gluconate, 20 TEA-Cl, 10 HEPES, 5 Na2-phosphocreatine, 4 MgATP, 0.3 GTP and 1 EGTA (pH 7.3). The patch pipette typically had a resistance of 4 MΩ, and the series resistance was an average of ∼14 MΩ. For rebound burst characterization, cells were held in a current clamp under a membrane voltage of about –85 to –50 mV (in 5 mV increments) via a constant current injection. Rebound bursting was determined following a 500 ms, –0.5 nA current step, as previously described.^16^ To determine T-currents, responses to different hyperpolarizing steps from –80 mV holding potential (500 ms, ranging from –110 to –50 mV) were recorded in the voltage-clamp configuration.

### *In situ* hybridization

We conducted the *in situ* hybridization (ISH) as previously described.^17^ Briefly the sections were treated with proteinase K (40 µg/ml, Roche, Germany) for 30 min and then they were acetylated. We generated digoxigenin (DIG)-labelled complementary RNA probes for three types of T-type calcium channels: *Cacna1g* (Cav3.1), *Cacna1h* (Cav3.2) and *Cacna1i* (Cav3.3). DIG-RNA probes were hybridized overnight at 63°C. DIG-RNA probes were reacted with an alkaline phosphatase-conjugated anti-DIG antibody (1:5000 dilution, Roche, Germany), and visualized with Nitro-Blue Tetrazolium Chloride/5-bromo,4-chloro,3-indolyl phosphate (Roche, Germany). Images were obtained with an All-in-one Fluorescence Microscope (BZ-X700, Keyence).

### RNA extraction and quantitative RT-PCR

Total RNA was extracted from cortices and thalami of PV-ArchT mice fed normal chow (*n* = 3 mice) and PV-ArchT mice fed doxycycline (DOX) chow (*n* = 3 mice). Mice were euthanized by cervical dislocation, the brains were removed from the skulls and the dissected cortex or thalamus was immediately lysed in Trizol reagent (Thermo Fisher Scientific, USA). Total RNA was converted to cDNA using ReverTra Ace qPCR RT Master Mix with gDNA Remover (TOYOBO, Japan). For quantitative RT-PCR analysis, we used target-specific TaqMan probe and primers sets as follows: *Cacna1g* (Mm01299131_m1), *Cacna1h* (Mm00445382_m1) and *Cacna1i* (Mm01299033_m1) (Thermo Fisher Scientific). Results were normalized to *Gapdh* mRNA (Mm03302249_g1) levels. The PCR was performed using the StepOne Real-Time PCR System (Thermo Fisher Scientific).

### DOX administration

DOX (doxycycline hyclate; Sigma, St Louis, MO, USA) was administered to mice in chow (CE-2, CLEA) containing 100 mg/kg DOX.

### Statistical analysis

Statistical analyses were performed using Excel and MATLAB. Data are expressed as mean ± SEM. *t*-tests were used to test differences between means in two-group comparisons. One-way ANOVA was used to test differences between the mean values in each group with ages. *P* < 0.05 is considered statistically significant (*P < 0.05 and ***P < 0.01).

### Data availability

The data sets that support the current study are available from the corresponding author on reasonable request.

## Results

### ArchT-expressing transgenic mice exhibited EEG abnormalities

**Figure 1:**
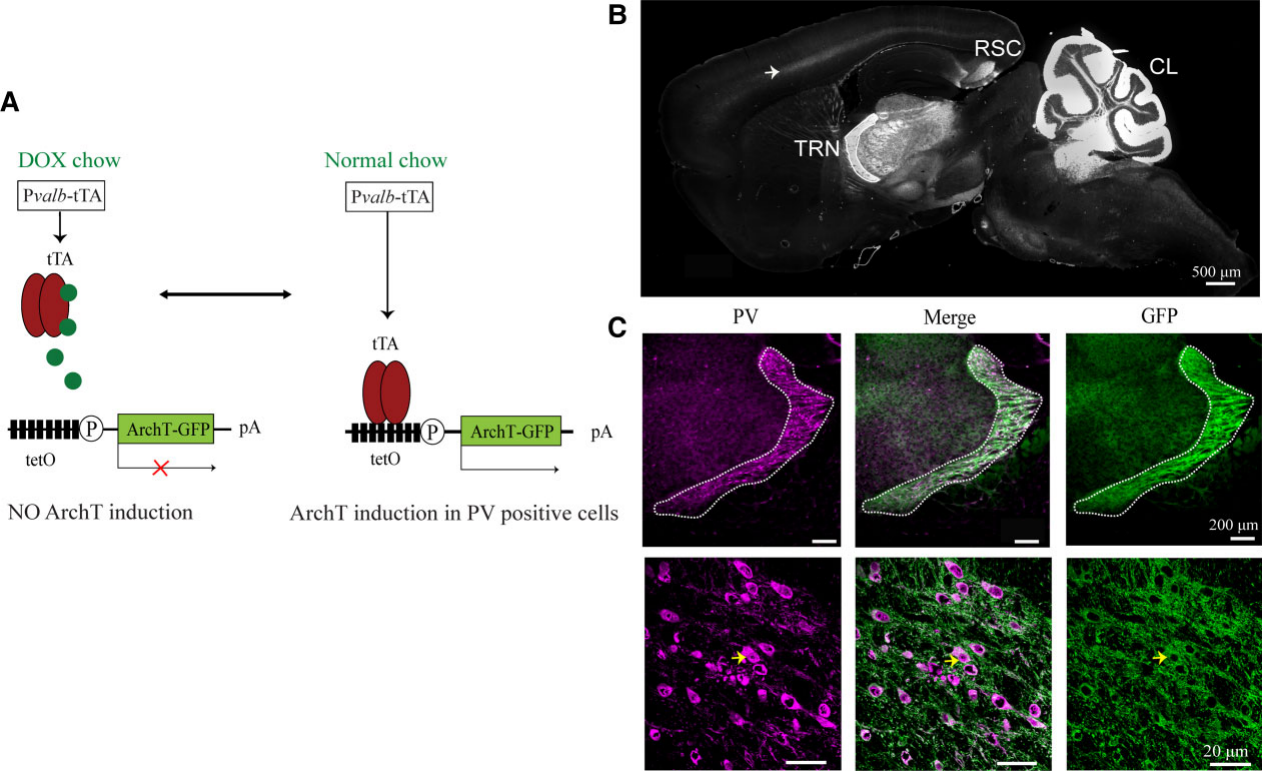
**ArchT-GFP expression in PV-positive cells using a Tet-Off system.** (**A**) ArchT-GFP was expressed by a Tet-Off system. tTA protein-induced ArchT-GFP expression in PV-positive neurons occurred in the absence of DOX. (**B**) Direct fluorescent image of a sagittal section from a PV-ArchT mouse fed normal chow (*n* = 3). TRN, thalamic reticular nucleus; CL, cerebellum; RSC, retrosplenial cortex. Scale bar, 500 μm. (**C**) Confocal images showing PV and GFP immunoreactivities in the TRN of a mouse fed normal chow (*n* = 3 mice). PV-positive cells (magenta) expressed ArchT-GFP (green). Yellow arrows confirm that PV-positive cells express GFP. *Top* scale bar, 200 μm; *bottom* scale bar, 20 µm.

We generated PV-ArchT mice, which contained the *Pvalb*-tTA and tetO-ArchT transgenes. In these mice, the tetracycline transactivator (tTA) protein was expressed under the control of the *Pvalb* promoter. In the absence of DOX, tTA is tethered to the tetracycline operator site (tetO), inducing the expression of the ArchT-GFP fusion protein (Fig. 1A). In mice fed normal chow (no DOX), ArchT-GFP was highly expressed in the TRN, cerebellum, accessory olfactory bulb and retrosplenial cortex, and moderately expressed in Layer 5 cortical neurons (arrow in Fig. 1B). Unexpectedly, ArchT-GFP was very weakly expressed, if at all, in cortical PV-positive interneurons. Within the TRN, 99 ± 0.5% of GFP-positive cells were PV-positive, and 72 ± 3.2% of PV-positive cells were GFP-positive (*n* = 3 mice, total 752 cells) (Fig. 1C).

**Figure 2:**
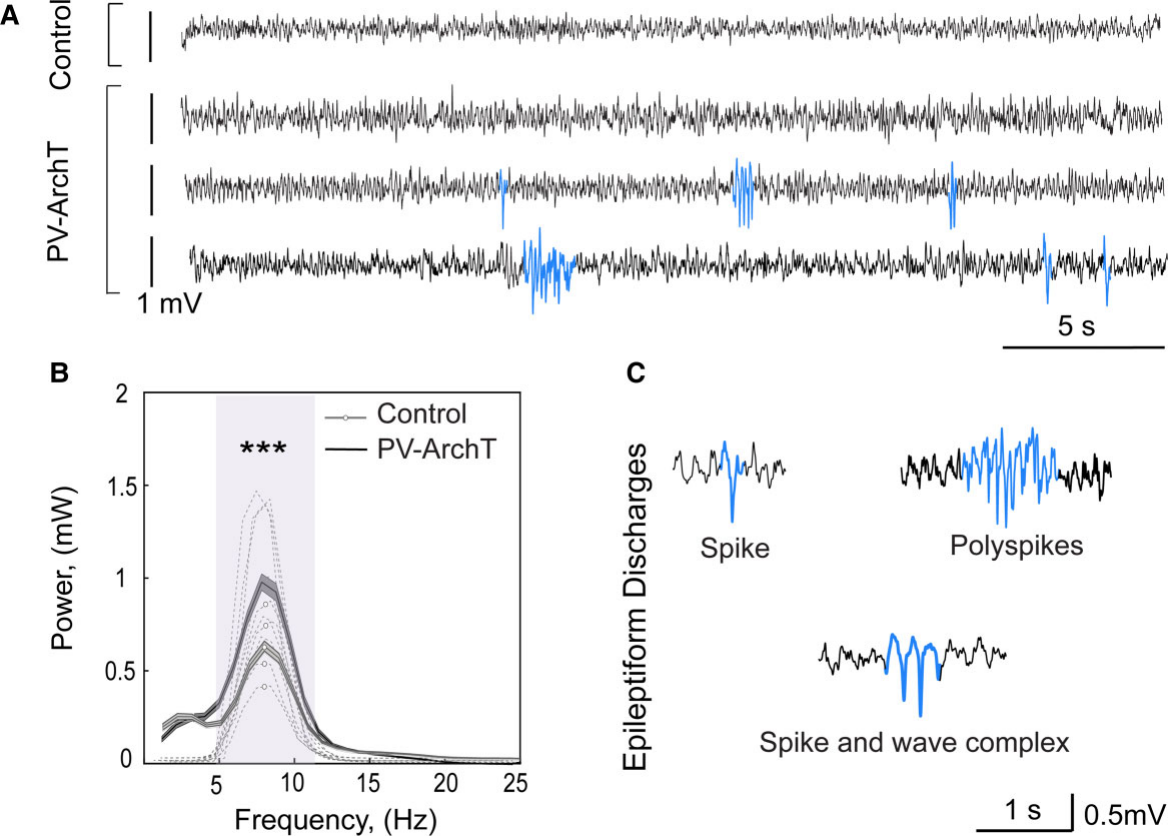
**PV-ArchT mice show abnormal EEG.** (**A**) Cortical EEG traces. *Top*: control; *bottom* three traces: PV-ArchT mice aged 2 months. EEG of PV-ArchT mice often included epileptiform discharges (blue traces). (**B**) Spectrograms of the power of EEG for controls and PV-ArchT. The dashed lines indicate the individual data, and the solid lines with shaded area indicate the mean ± SEM. PV-ArchT mice aged 2 months showed high theta power (4–11 Hz, purple highlighted, unpaired *t*-test, *t*_10_ = 2.799, *P* = 0.005). (**C**) Typical shapes of epileptiform discharges in PV-ArchT mice.

PV-ArchT mice developed normally in early life and did not show behavioural abnormalities such as gait disturbance or ataxia. However, PV-ArchT mice exhibited cortical EEG abnormalities at 2 months, the earliest time to start EEG measurement in our experimental system. These EEG abnormalities appeared without any light activation of the ArchT opsin, despite the light-dependent functions of ArchT. EEG abnormalities included a high-power theta rhythm (theta frequency band 4–10 Hz) (Fig. 2A and B) and epileptiform discharges (Fig. 2A and C). Epileptiform discharges observed in PV-ArchT mice were spikes (a sharp deflection), polyspikes (runs of more than two spikes) and spike-and-wave complexes (a pattern consisting of a spike followed by a slow wave) (blue traces in Fig. 2A and C).

**Figure 3:**
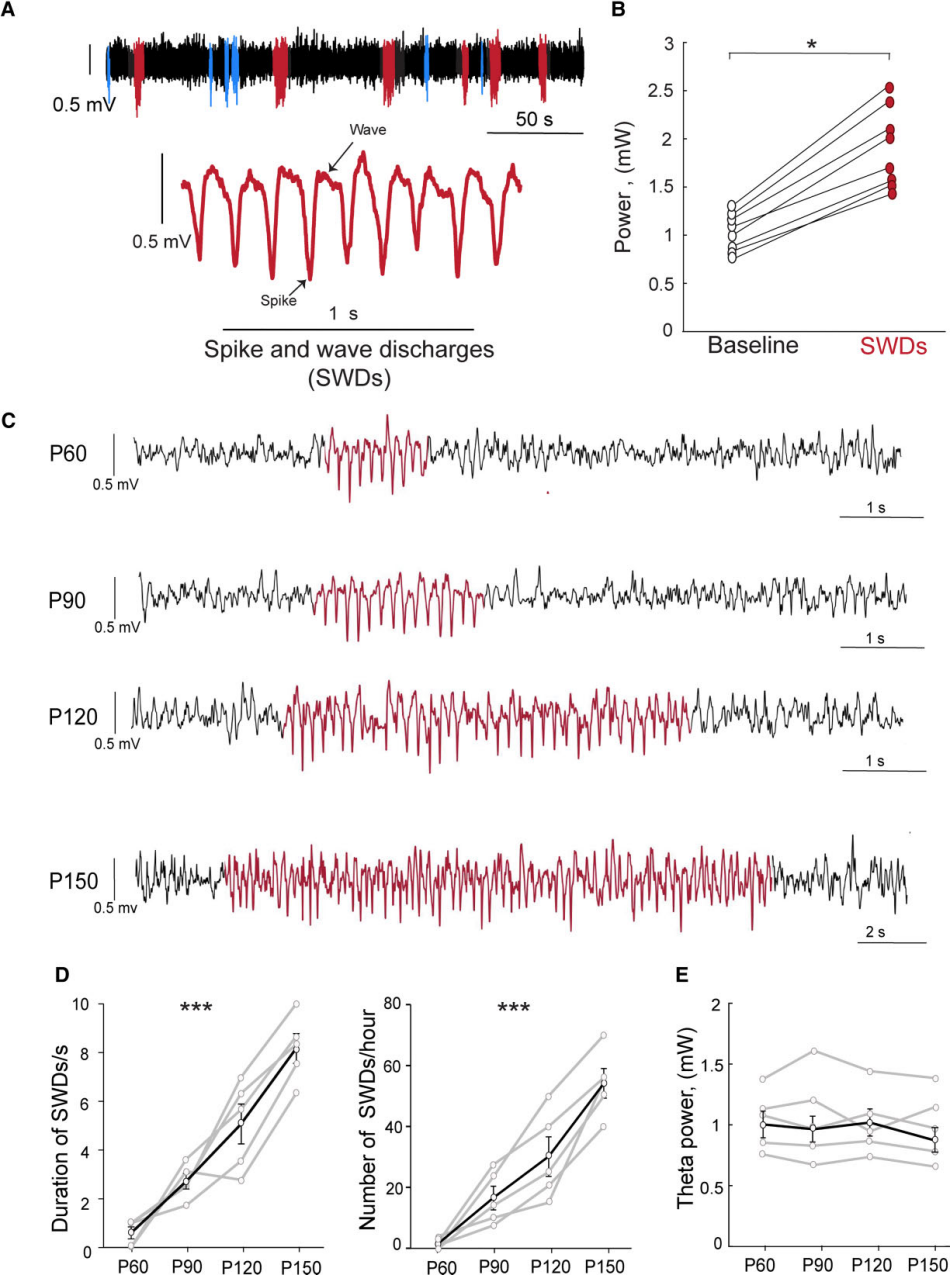
**Characterization of SWD in PV-ArchT mice.** (**A**) PV-ArchT mice aged >2 months exhibited SWDs (red traces) and other types of epileptiform discharges (blue traces). (**B**) The EEG power of SWD events was significantly higher than that of baseline (4–11 Hz, paired *t*-test, *t*_7_ = 2.32, *P* = 0.03). (**C**) Age-dependent shows the worsen of SWDs events (SWDs; red traces). (**D**) Line plots show a significant increase in SWD duration (left panel) and frequencies (right panel) with age from P60 to P150 [one-way ANOVA, duration: *n* = 5 mice; *F*(3,12) = 23, *P* = 2 × 10^−5^; frequency: *n* = 5 mice; *F*(3,12) = 30, *P* = 7 × 10^−6^] from P60 to P150. (**E**) Line plots show no changes of high theta power with age from P60 and P150 [one-way ANOVA, *n* = 5 mice: *F*(3,812) = 0.01, *P* = 0.999].

With age, the length and the frequency of spike-and-wave complexes increased. To quantify the degree of epileptiform discharges, we first defined the SWD as a repetitive spike-and-wave lasting >0.5 s and ranging from 7 to 10 Hz (Fig. 3A). The theta power of SWDs was significantly higher than that of heightened baseline EEG (Fig. 3B); thus, it was relatively easy to identify SWD events despite the basal high-power theta rhythm of PV-ArchT mice. Based on these criteria, we detected the SWD event at various ages. The duration and the number of SWDs increased with age (Fig. 3C and D), indicating the age-dependent progression of the SWD occurrence. However, we did not detect any further increase in theta power with age (Fig. 3E).

### PV-ArchT mice displayed an absence seizure phenotype

**Figure 4:**
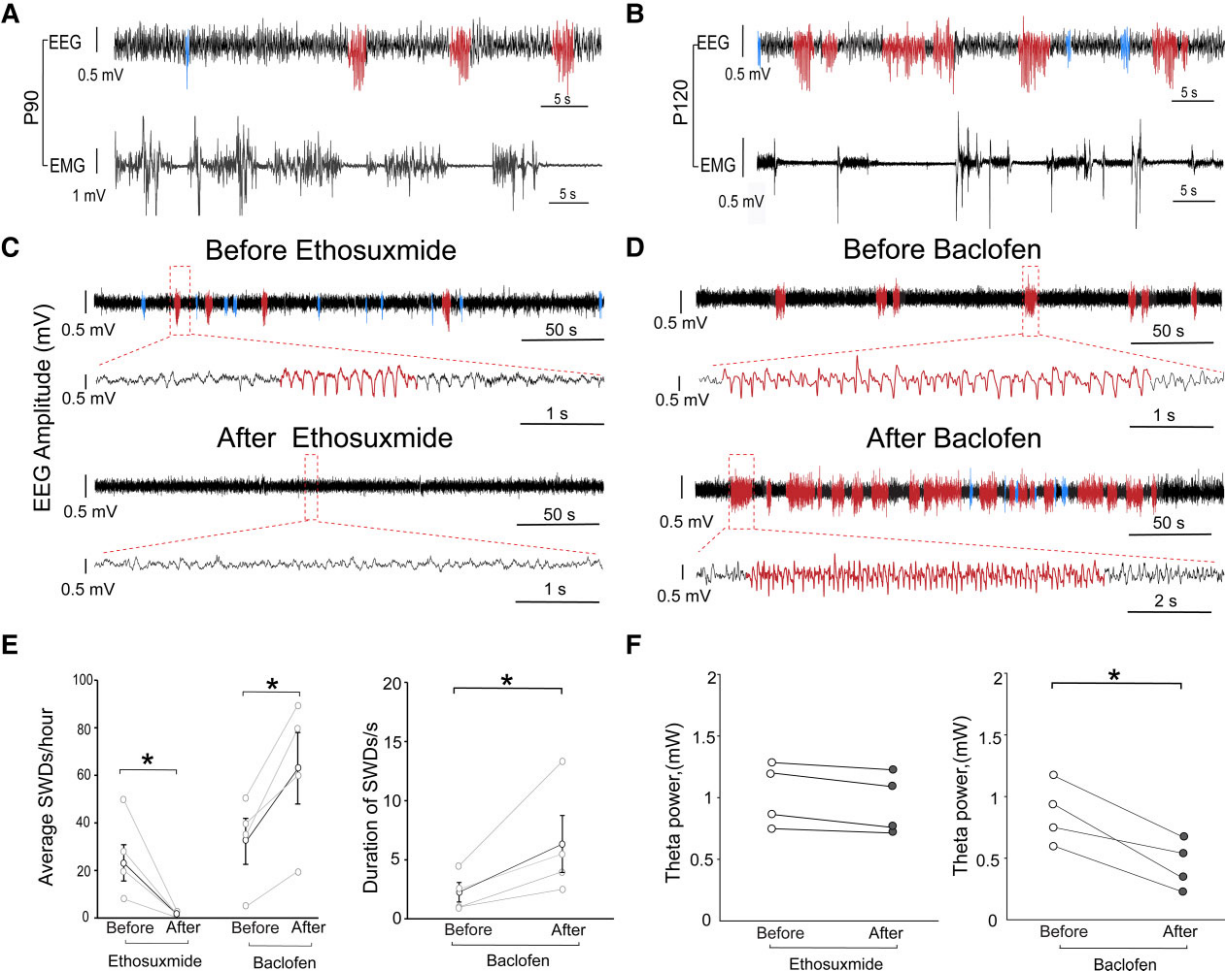
**PV-ArchT mice exhibited an absence seizure phenotype.** Cortical EEG and EMG in PV-ArchT mice at P90 (**A**) and P120 (**B**). Both ages show SWDs (red traces) and other types of epileptiform discharges (blue traces). SWD events (98.8% from 85 SWDs at P90 and 91.5% from 150 SWDs at P120) were associated with low EMG amplitude. (**C**) Before (*top*) and after (*bottom*) administering ethosuximide to PV-ArchT mice (3–6 months). Ethosuximide abolished SWDs (before 23 ± 7.6/h versus after treatment 1.8 ± 0.7/h, *n* = 4 mice). (**D**) Before (*top*) and after (*bottom*) baclofen administration. Baclofen increased the frequency (before 32 ± 9.7/h versus after treatment 63 ± 15/h, *n* = 4 mice). (**E**) Line plots (left panel) show a significant decrease and an increase in SWD frequency after ethosuximide and baclofen administration, respectively (ethosuximide: paired *t*-test, *t*_3_ = 2.9, *P* = 0.03; baclofen: paired *t*-test, *t*_3_ = 2.4, *P* = 0.02). A line plot (right panel) shows significantly increased duration after baclofen (2.3 ± 0.8 versus 6.2 ± 2.4 s, paired *t*-test, *t*_3_ = 2.4, *P* = 0.04). (**F**) Line plots (left panel) show no changes of the theta power after ethosuximide (paired *t*-test, *t*_3_ = −1.54, *P* = 0.164). The line plots (right panel) show significantly decreased high theta power after baclofen (paired *t*-test, *t*_3_ = 2.21, *P* = 0.038).

PV-ArchT mice showed intermittent episodes of behavioural arrest. To determine whether the behavioural arrest was associated with SWD, we recorded EEG and EMG with video monitoring. During an attack, the mouse stopped its ongoing behaviour and did not respond to external stimuli such as sound or light (Video 1) and the behavioural arrest was always associated with SWDs. Although EMG amplitudes were very low during attacks (Fig. 4A and B), mice kept their postures and did not fall. Once the attack terminated, the mouse’s behaviour and EMG amplitude were restored. These behaviours were consistent with an unconscious phenotype seen in human absence seizures. The nature of SWDs in humans (3 Hz, more than a 10 s duration)^3,4^ is distinct from that observed in rodent models (>4 Hz, short duration)^18^ and that in our model. However, both the EEG/EMG characteristics and the behaviour mimic absence seizure in PV-ArchT mice, and this model can be considered to have face validity.

**Video 1:** **PV-ArchT mice exhibited SWDs.** The mouse exhibited a behavioral arrest associated with SWDs. The black arrow indicates the end of SWDs, and the mouse resumed the behavior.

To examine the predictive validity of this absence seizure model, we administered ethosuximide (100 mg/kg intraperitoneal injection), the first-line medication for absence seizure in humans,^19^ to PV-ArchT mice. Ethosuximide had an acute effect on the frequency of SWDs and abolished SWDs 30 min after treatment (Fig. 4C and E). Therefore, this model also exhibits predictive validity. It is known that baclofen, a GABAB agonist, worsens EEG abnormalities in other validated absence seizure rodent models.^20,21^ We administered baclofen (20 mg/kg, intraperitoneal injection) to PV-ArchT mice to test whether the baclofen treatment could exacerbate the EEG phenotype. As expected, 30 min after baclofen treatment, the frequency and the duration of SWDs increased (Fig. 4D and E). To examine the pharmacological response to high-power theta rhythm, we calculated theta power before and after drug administration. Ethosuximide did not reduce theta power (Fig. 4F), but baclofen rather reduced it (Fig. 4F), suggesting that therapeutic and detrimental drug mechanisms in SWDs are distinct from those in high-power theta rhythm.

Taken together, expression of the opsin ArchT in PV-positive neurons caused abnormal brain waves, indicating that ArchT expression—even without light activation—had a toxic effect on PV-positive cells. During the epileptic attack, mice stopped all behaviour and showed unconsciousness, which is consistent with an absence seizure phenotype in humans. In addition, PV-ArchT mice satisfied traits of predictive validity as an absence seizure animal model.

### ArchT expression in PV-positive TRN neurons induced cortical SWDs

**Figure 5:**
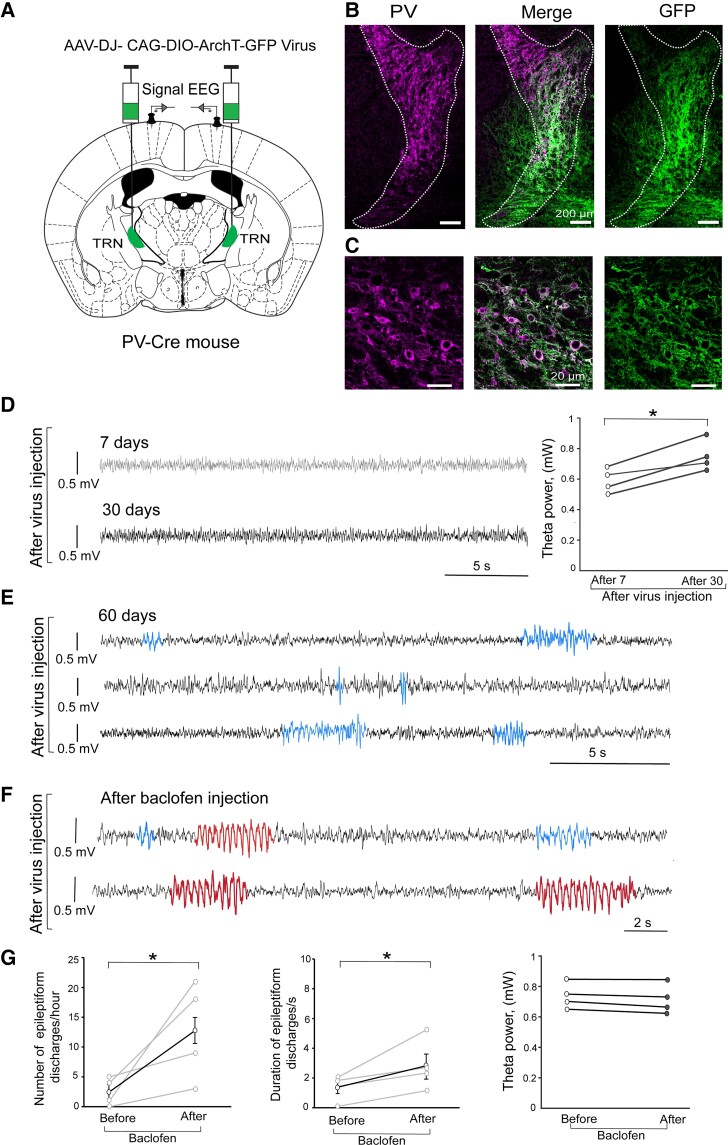
**ArchT expression in the TRN-induced SWDs.** (**A**) A strategy of ArchT-GFP expression only in the TRN. PV-Cre mice were injected with AAV carrying CAG-DIO-ArchT-GFP, resulting in ArchT-GFP expression in TRN PV-positive cells (*n* = 4 mice). (**B**) AAV injection targeted the middle portion of the dotted line of TRN. Scale bar, 200 μm. (**C**) Confocal images showing the localization of ArchT-GFP signals (green) and PV signals (magenta). Scale bar, 20 μm. (**D–F**) Cortical EEG waveforms after the virus injection. (**D**) Cortical EEG traces at 7 days (*top* grey) and 30 days (*bottom* black) after virus injection. The line plots show increased theta power 30 days after virus injection (paired *t*-test, *t*_3_ = −2.34, *P* = 0.019). (**E**) Cortical EEG at 60 days after virus injection show epileptiform discharges (blue traces). (**F**) Baclofen administration significantly induced typical SWDs (red traces) with epileptiform discharges (blue traces). (**G**) Line plots show increased frequency (left panel) and duration (middle panel) of epileptiform discharges after baclofen administration (frequency: before 2.5 ± 1.2/h versus after 13 ± 4.1/h, paired *t*-test, *t*_3_ = 2.35, *P* = 0.04; duration: before 1.7 ± 0.6 versus after 2.7 ± 0.9 s, paired *t*-test, *t*_3_ = 2.35, *P* = 0.04); and right panel shows no changes of high theta power (paired *t*-test, *t*_3_ = 1.9, *P* = 0.265).

Cortico-thalamo-cortical circuit dysfunction is considered to cause absence seizures.^5,6^ We addressed the direct role of the TRN in SWD pathogenesis by using viral delivery of ArchT. We injected AAVDJ-CAG-DIO-ArchT-GFP bilaterally into the middle part of the TRN in adult *Pvalb*-Cre mice (Fig. 5A). We confirmed ArchT expression by IHC; ArchT-GFP was labelled only in PV-positive neurons (68.0 ± 10.1% in PV-positive neurons in the target area, Fig. 5B and C). After mice recovered from the viral injection, we made weekly EEG recordings. We first recorded cortical EEG after 1 week as baseline control (grey trace in Fig. 5D). We found significant higher theta power 1 month after AAV injection (black trace in Fig. 5D, and right panel in Fig. 5D). We then observed epileptiform discharges 2 months after AAV injection (blue traces in Fig. 5E). The frequency of epileptiform discharges was 2.5 ± 1.2/h (*n* = 4 mice) and the duration ranged 1.3 ± 0.4 s (median 1.6 s). However, we did not observe a typical SWD.

The baclofen challenge induced the typical SWD (red traces in Fig. 5F) and increased the frequency of epileptiform discharges and their duration (Fig. 5F), while theta power did not change significantly after baclofen (Fig. 5F). These data indicate that AAV-mediated ArchT expression in TRN PV-positive neurons are sufficient to induce cortical epileptiform discharges and that such discharges are continuous with SWDs.

### ArchT expression impaired hyperpolarization-induced rebound excitation in TRN PV-positive neurons

While ArchT has been widely used in optogenetics,^22^ we previously found that ArchT expression itself in oligodendrocytes, a glial cell type in the brain, caused dysmyelination.^23^ Our previous and current results indicate that ArchT has toxic functions in a cell type-dependent manner.

To address how ArchT expression alters the basic cellular functions of TRN PV-positive neurons, we conducted slice physiology experiments and examined membrane properties. We prepared acute slices from PV-ArchT (*n* = 3 mice, 20 cells) and PV-YC (*Pvalb*-tTA::tetO-YC) (*n* = 3 mice, 10 cells) juvenile mice where TRN PV-positive cells were labelled by GFP and yellow fluorescent protein, respectively. YC is a genetically encoding a calcium indicator and we used PV-YC mice as the control. Resting membrane potential and input resistance were comparable between ArchT-expressing and control (PV-YC) cells (−77.1 ± 0.4 versus −77.5 ± 0.5 mV, *t*_4_ = 0.5, *P* = 0.6; 180.8 ± 40.8 versus 157.3 ± 9.2** **MΩ, *t*_4_ = 0.6, *P* = 0.6; data are stated in the order of PV-ArchT versus PV-YC), and other important metrics of intrinsic excitability (including membrane time constant, membrane capacitance and AP threshold; Supplementary Table 1).

**Figure 6:**
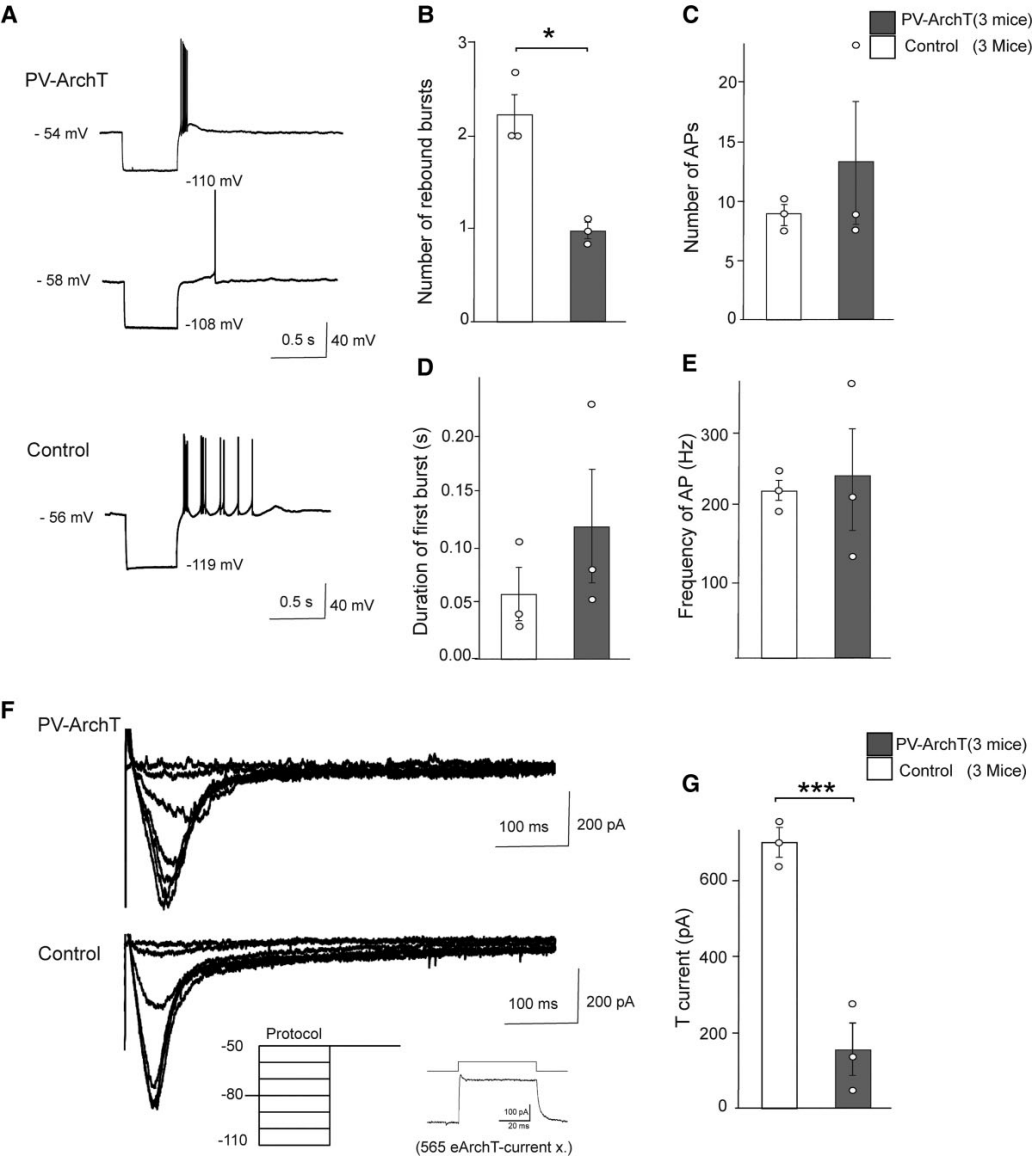
**ArchT expression in the TRN impaired rebound bursting and T-current.** (**A**) Representative traces of rebound burst firing in PV neurons following hyperpolarization from PV-ArchT mouse brain slices (*top trace*, 13 cells with single bursts; *middle trace*, 4 cells did not evoke burst firing) and from controls (*bottom trace*, 10 cells evoked burst firing). The data were from three PV-ArchT mice and three controls. Neurons were injected with negative current (−0.5 nA) to induce hyperpolarization to −112 mV. Scale indicates 0.5 s and 40 mV. (**B**) Number of rebound bursts after hyperpolarization (10 cells from 3 control and 20 cells from 3 PV-ArchT mice). (**C**) Number of action potentials (APs) in the first burst (10 cells from control and 20 cells from PV-ArchT). (**D**) Duration required for first bursts to evoke action potentials (10 cells from 3 mice control and 16 cells from 3 PV-ArchT). (**E**) Frequency of APs from first bursts. (**F**) T-current was evoked by hyperpolarizing steps (500 ms, ranging from −110 to −50 mV, with 10 mV increments) in PV-ArchT (*top trace*) and control (*bottom trace*). Inset shows an example trace of the light-evoked response from a PV-ArchT neuron at a holding potential of −80 mV (**G**) T-current amplitude. Data are shown as mean ± SEM. *P < 0.05 and ***<0.01, unpaired *t*-test comparing PV-ArchT and control groups.

TRN PV neurons show hyperpolarization-induced rebound excitation, and this type of excitation generates burst firing in cells.^24,25^ We injected negative current into cells and examined the rebound excitation. In controls (PV-YC), all the cells we tested (10 cells from 3 mice) exhibited after-hyperpolarization-evoked bursts of action potentials: four cells with three bursts (Fig. 6A, bottom), four cells with two bursts and two cells with a single burst. In PV-ArchT cells (20 cells from 3 mice), the number of bursts was significantly impaired compared with controls (Fig. 6B, 0.99 ± 0.08 for PV-ArchT versus 2.22 ± 0.2 for controls, *t*_4_ = −5.2, *P* = 0.02): 1 cell with 3 bursts, 2 cells with 2 bursts and 13 cells with single bursts (Fig. 6A, top), and 4 cells did not evoke any bursts (Fig. 6A, middle). The number of action potentials on the first burst (Fig. 6C), the duration of first bursts (Fig. 6D) and the frequency of action potentials in the first burst (Fig. 6E) were comparable between the two groups.

Rebound excitation of TRN PV cells is mediated by T-type calcium channel current.^26^ We measured T-type calcium channel current in both PV-ArchT and PV-YC cells. ArchT-expressing TRN PV cells possessed significantly lower peak current than controls (Fig. 6F) (155 ± 68 pA for PV-ArchT versus 699 ± 36 pA for controls, *t*_4_ = −7, *P* = 0.006: Fig. 6G). Collectively, these data indicated that T-type calcium channel current was impaired by ArchT expression in TRN PV-positive cells.

### Thalamic hyperactivity was synchronized to cortical SWDs in PV-ArchT mice

We now regard PV-ArchT mice as a model of absence seizures according to face (SWDs) and predictive (effectiveness of ethosuximide) validities. Ethosuximide is a T-type calcium channel blocker; however, ArchT expression in TRN PV-positive neurons was the primary cause of T-current impairment in TRN PV-positive neurons. To reconcile TRN dysfunction with ethosuximide effectiveness, we investigated whether there was hyperactivity of the thalamus. We measured thalamic activity by extracellular recording in the anteroventral thalamus (Supplementary Fig. 1A) and examined whether thalamic hyperactivity was synchronized with cortical SWDs in PV-ArchT mice. Thalamic LFPs with high amplitudes are associated with cortical SWDs and low EMG amplitudes (Supplementary Fig. 1B and C). The dominant frequency of thalamic LFPs, ranging from 7 to 10 Hz, is the same as that of cortical SWDs. The degree of coincidence of SWD timing in the cortex and the thalamus was high: 87% of thalamic hyperactivity coincided with cortical SWDs, and all cortical SWDs coincided with thalamic hyperactivity.

**Figure 7:**
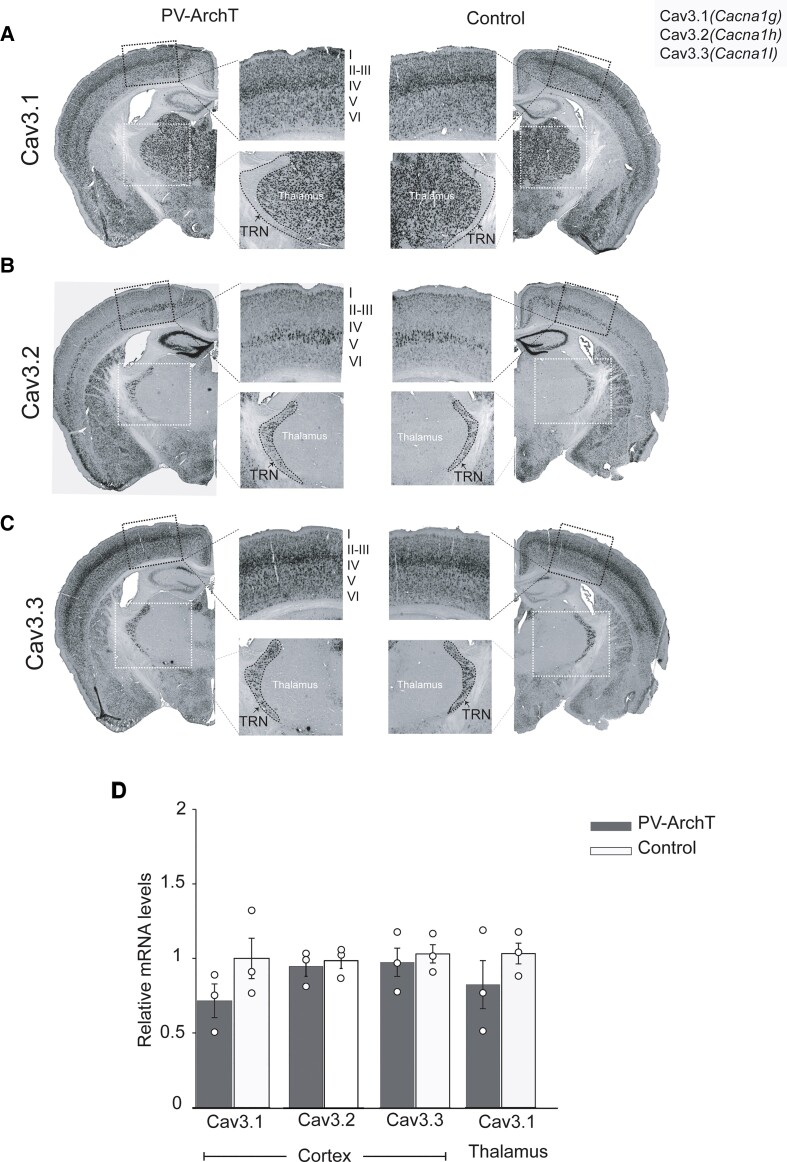
**Cells expressing T-type Ca^2+^ channel mRNA transcripts in PV-ArchT mouse brains compared with controls.** ISH of T-type Ca^2+^ channel transcripts is shown. Left, PV-ArchT; right, control. (**A**) Cav3.1 (*Cacna1g*) was expressed in all cortical layers and in the thalamus, but not in the TRN. (**B**) Cav3.2 (*Cacna1h*) was strongly expressed in Layer V and in the TRN. (**C**) Cav3.3 (*Cacna1i*) was found in all layers of the cortex and in the TRN. The inset of the black square shows the magnification of the cortex, and the inset of the white square shows the magnification of the thalamus and TRN. Note that the expression of T-type Ca^2+^ channel transcripts did not show difference in the cortex, thalamus and TRN comparing PV-ArchT with the control (*n* = 4 mice). (**D**) Graphs show transcript levels, as measured with quantitative RT-PCR, of the three types of T-type Ca^2+^ channels in the cortex and thalamus. No significant changes between PV-ArchT and control, respectively (Cav3.1; 0.72 ± 0.11 versus 1 ± 0.14; Cav3.2; 0.96 ± 0.07 versus 1 ± 0.05; Cav 3.3; 0.94 ± 0.09 versus 1 ± 0.05; Cav3.1 of the thalamus 0.79 ± 0.16 versus 1 ± 0.07, *n* = 3 mice for each group). Data are shown as mean ± SEM.

T-currents are mediated by voltage T-type calcium channels (Ca_v_T), which are encoded by *Cacna1g* (*Cav3.1*), *Cacna1h* (*Cav3.2*) and *Cacna1i* (*Cav3.3*). We next examined whether ArchT expression affected the transcript levels of T-type calcium channels. We examined the change in mRNA distribution for each gene by ISH. *Cacna1g* was expressed by the cortex and the thalamus (Fig. 7A), and *Cacna1h* and *Cacna1i* transcripts were expressed in the cortex and the TRN of controls (Fig. 7B and C), as reported by the Allen Brain Atlas. ArchT expression did not change the mRNA distribution pattern. In particular, ArchT expression did not induce *Cacna1h* and *Cacna1i* mRNA in the thalamus.

We used quantitative RT-PCR to detect mRNA expression changes in the thalamus and the cortex. We did not detect any increase in Cav mRNA levels (Fig. 7D). These negative data indicate that alterations in Cav mRNA expression levels could not account for the ethosuximide-sensitive component of cortical and thalamic hyperactivity. Taken together, ArchT expression in TRN PV-positive cells resulted in both thalamic and cortical hyperactivation. This hyperactivation was normalized by a Cav blocker, ethosuximide, but we did not observe a Cav gain of function at the transcriptional level.

### SWDs were dose- and time-dependently induced by ArchT-GFP expression

PV-ArchT mice might serve as a novel animal model of absence seizures. The dosage and timing of ArchT expression in our transgenic mouse are DOX-controllable. Therefore, we asked the following questions: (i) Is the childhood-onset absence seizure phenotype rescued by the removal of toxic ArchT? and (ii) does induction of ArchT in adulthood produce the absence seizure phenotype?

**Figure 8:**
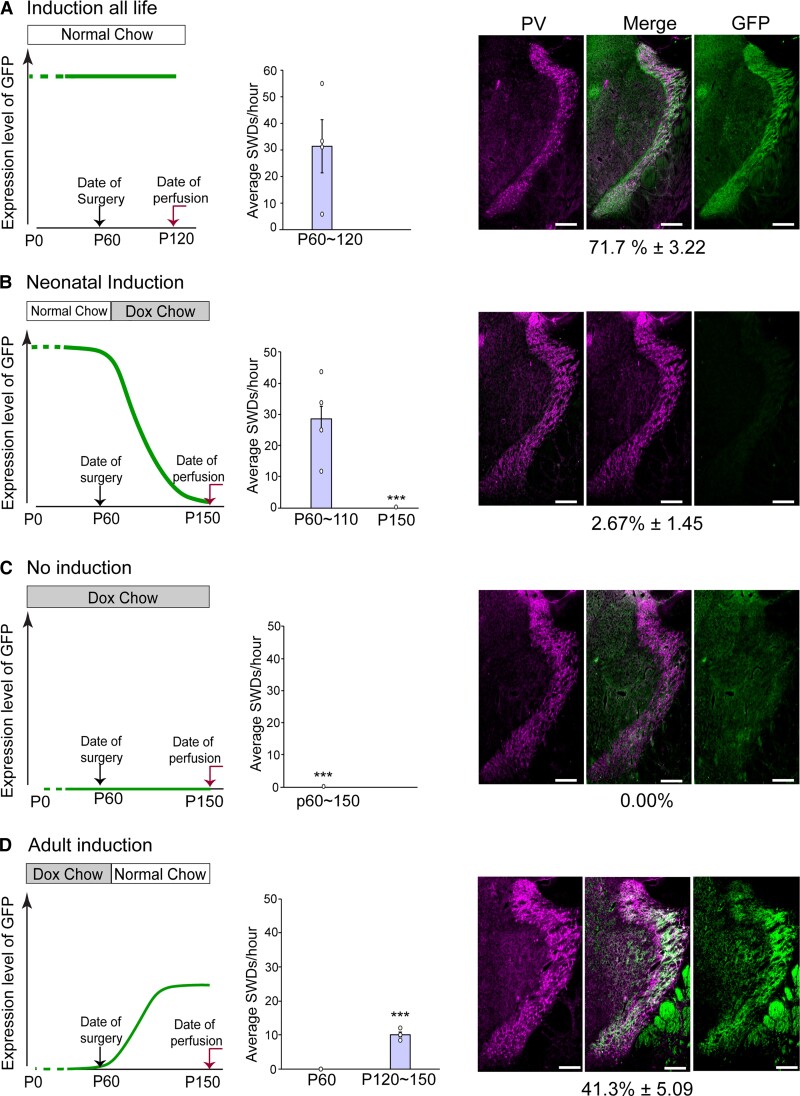
**SWDs were dose- and time-dependently induced by ArchT.** Summary graphs (left) showing the feeding regimen (DOX or normal chow) and the corresponding level of ArchT-GFP expression; charts (middle) presenting the average SWDs/h; and IHC images (right) of the TRN showing GFP (green, representing ArchT) in PV neurons (magenta) and the merged image of the two, scale bar, 200 μm. (**A**) Mice under a normal chow their entire lives (*n* = 4 mice). (**B**) Mice with neonatal induction of ArchT (27 ± 3.8 times/h, P60, *n* = 4 mice). (**C**) Mice under DOX chow their entire lives (*n* = 3 mice). (**D**) Mice with adult induction of ArchT (9.1 ± 0.9 times/h, *n* = 3 mice). Data are shown as mean ± SEM. *P < 0.05 based on paired *t*-tests comparing two time points.

To address the reversibility of the disease phenotype, we switched the diet of PV-ArchT mice from normal chow to DOX-containing chow at P60 and maintained the mice on this DOX chow thereafter. DOX administration turned off ArchT-GFP induction (Fig. 1A) and the population of GFP/PV double-positive cells was markedly reduced (2.7 ± 1.5% at P150; Fig. 8B) compared with that in mice fed by normal chow (72 ± 3.2%; Fig. 8A). Along with the reduced ArchT levels, within-subject SWDs disappeared gradually 2 months after DOX initiation, demonstrating that the disease phenotype is reversible in PV-ArchT mice.

DOX completely suppressed ArchT in mice receiving DOX throughout their whole lives (Fig. 8C), and unsurprisingly, ArchT was never expressed in the TRN and SWDs even at 5 months of age in PV-ArchT mice. In this same group of mice, when we expressed ArchT in PV-positive cells by switching to normal chow at P60, ArchT expression gradually increased and the population of GFP/PV double-positive cells reached 41 ± 5.1% (*n* = 3 mice) at P150. In the DOX on-to-off regimen (Fig. 8D), after 2 months of induction, we observed typical SWDs (red traces, Supplementary Fig. 2A). Although tTA-mediated ArchT induction (Fig. 8D) and AAV-mediated ArchT expression (Fig. 5) are different gene expression systems, these data lead to the same conclusion that adult ArchT expression in TRN PV-positive cells induced SWDs.

## Discussion

This study describes a new transgenic mouse, PV-ArchT, that met the validity requirements (face and predictive) of an animal model of absence seizures. This mouse model exhibits the ability to switch SWDs on and off at different ages under the control of a DOX diet. It is generally understood that the mechanism of SWDs involves abnormalities between the cortex and thalamus, and the TRN is considered a pacemaker that propagates the seizures. However, our study supports the hypothesis that TRN-centred dysfunction *per se* is sufficient to induce SWDs and that the TRN is not simply a modulator. On the other hand, although rebound bursts of the TRN are important to propagate SWDs, PV-ArchT mice exhibited SWDs with impaired in burst firing. Finally, our experiments supported the acute effectiveness of ethosuximide on SWDs, possibly through action in the cortex and thalamus since T-current in the TRN was impaired. In humans, the mechanism of how SWDs are abolished after puberty is unclear and thus, our mouse model will be useful in future studies seeking to understand this phenomenon.

### The PV-ArchT mouse is a new model of absence seizure, fulfilling criteria for face and predictive validities

Our new PV-ArchT transgenic mouse exhibited EEG/EMG patterns associated with the behavioural arrest and the mimicked absence seizure-like SWDs. These findings indicate that PV-ArchT mice met the validity criteria required for a successful animal model of human disease.^27^

In this section, we discuss how the face and predictive validities are fulfilled by PV-ArchT mice and propose that they can serve as a new animal model of absence seizure. Face validity for absence seizure requires that seizures in the mouse model show similar EEG signs and behavioural features as seizures in humans, while predictive validity requires that the pharmacological effects are similar between seizures in the mouse model and humans. PV-ArchT mice exhibited spontaneous, cortical 7–11 Hz SWDs that were bilateral and associated with behavioural arrest. Also, ethosuximide had a significant effect that decreased SWDs events of PV-ArchT mice. Despite these validity-based similarities of PV-ArchT mice and human seizures, there are some differences compared with human absence seizures. In humans, SWDs are ∼3 Hz, but in PV-ArchT mice, the SWD frequency is 7–11 Hz. Moreover, absence seizure is characteristically a childhood disease, which may disappear after mid-adolescence in humans, but in PV-ArchT mice, SWDs are controlled at any age by expressing ArchT in a DOX-controllable manner.

Our PV-ArchT mice are similar to two existing genetic rat models that are validated for absence seizure^28^: Wistar Albino Glaxo Rijswijk (*WAG-Rij*) and Genetic Absence Epilepsy Rat from Strasbourg (*GAERs*) rats. Both the PV-ArchT mice and rats share the feature of 7–10 Hz SWDs associated with behavioural arrest. But in the genetic *WAG-Rij* and *GAERs* rat models, SWDs sometimes associated with abnormal behaviours such as head-tilting and vibrissa- and eye-twitching. Moreover, the onset of SWDs differed between the rat models. SWDs occur at the ages of 30, 140 and 60 days for *GAERs* rats, *WAG/Rij* rats and PV-ArchT mice, respectively. As we mentioned before, SWDs in PV-ArchT mice are controllable but, in the rat, genetic models, they persist throughout life.

Several spontaneous mutant mice exhibit SWDs and are considered absence seizure model mice.^29^ Those mutants include the so-called tottering (*Cacna1a^tg^*), ducky (*Cacna2d2^du^*), stargazer (*Cacng2^stg^*), lethargic (*Cacnb4^lh^*) and slow-wave epilepsy (*Slc9a1^swe^*) mice. All mutants start to exhibit ataxia and absence seizure from P14 to 35, typically with a frequency of 5–7 Hz,^18,30^ except for the slow-wave epilepsy mice (1–3 Hz).^31^ PV-ArchT mice strongly expressed ArchT in the cerebellar Purkinje cells (Fig. 1); however, the cerebellar function seemed to be spared and they did not show ataxia. The absence of ataxia in PV-ArchT mice is distinct from the spontaneous mutant mouse models. As we mentioned before, SWDs in PV-ArchT mice are controllable, but, in both rat and mouse genetic models, they persist throughout life.

### Do cortical SWDs require the rebound excitation of TRN PV-positive neurons?

Rebound excitation in TRN neurons is mediated by T-type Ca^2+^ channels.^32,33^ Three isoforms of T-type Ca^2+^ channels are expressed throughout the thalamo-cortico-thalamic network: *Cav3.1*, *Cav3.2* and *Cav3.3*, with *3.2* and *3.3* being dominant in the TRN.^34^ These isoforms are considered to cause SWDs.^35^ Human studies reported that a mutation of TRN-expressing T-type Ca^2+^ channels is associated with absence seizures.^36^ A *Cav3.2* mutant was observed in Chinese patients suffering from absence seizures. The cellular function of the *Cav3.2* mutant provides increasing the activity of T-current. To study the *Cav3.3* isoform, *Cav3.3^−/−^* mice were used and it was found that they had a complete loss of burst firing in the TRN, indicating that SWDs were abolished.^37,38^ In other words, T-current plays a significant role in the induction of SWDs by rebound burst firing.

Lee’s group did not find any evidence that burst firing in the TRN is required for the generation of SWDs. They generated double knockout mice of *Cav3.2* and *Cav3.3* and demonstrated the complete abolishment of rebound bursts in TRN neurons. Even when the TRN had a loss of rebound bursts, the systemic injection of gamma-butyrolactone (GBL, a pharmacological animal model of absence seizure) still induced SWDs. Moreover, GBL-induced SWDs were highly sensitive to the T-type Ca^2+^ channel blocker, ethosuximide.^35^ Instead, they found increased tonic firing in TRN neurons due to the loss of rebound bursts and proposed that tonic firing in TRN neurons may mediate the TC rhythm more efficiently.

Our PV-ArchT mice share a similarity with the *Cav3.2* and *Cav3.3* double knockout phenotype. PV-ArchT mice showed an impairment, but not a complete loss, of rebound bursts and showed spontaneous SWD formation (but not GBL-induced SWDs), and ethosuximide treatment abolished these spontaneous SWDs. We did not examine tonic firing in TRN neurons of PV-ArchT mice, and therefore, we cannot propose the same pathophysiological mechanism leading to spontaneous SWD generation as proposed for the *Cav3.2* and *Cav3.3* double knockout mice. However, the shared therapeutic effects of ethosuximide on SWDs may give us some hints regarding the mechanism of ethosuximide, as well as provide insights into the development of new drugs for the treatment of absence seizure. We examined T-type Ca^2+^ channel mRNA distribution and levels and found no alteration in either by ArchT expression (Fig. 7), indicating that PV-ArchT mice did not exhibit compensatory responses at the transcriptional level to the impaired rebound bursts. Therefore, it will be necessary to examine post-translational alterations of T-type Ca^2+^ channels such as protein levels or membrane trafficking to elucidate the pathophysiology of SWD generation in line with ethosuximide effectiveness.

The mechanisms of increased SWDs with reduced TRN neuronal burst firing and reduced T-current in TRN PV-positive neurons remain unclear. This suggests a mechanism other than increased T-current may account for the SWD generation. The absence seizure phenotype induced by ArchT expression in the TRN provided a new perspective; however, we note that a single study cannot fully elucidate the entire mechanism. One plausible idea is that impaired function of inhibitory neurons could remove inhibition from other neurons, thereby causing them to become hyperexcitable and hypersynchronized. This secondary hyperexcitability could include both other inhibitory neurons (e.g. other TRN neurons, which could drive abnormal rhythms in thalamic relay neurons), as well as excitatory neurons (e.g. in the cortex, disinhibited by the pathology of ArchT-expressing cortical neurons). Future studies inspired by the idea above will be able to clarify the mechanisms underlying absence seizure.

### Time-controllable ArchT expression

The absence seizure-like phenotype persists throughout life in existing genetic animal models. In humans, absence seizures usually start between 4 and 15 years of age and often disappear naturally by mid-adolescence. The reason why absence seizures naturally regress is not totally known, and therefore, animal models mimicking this regression would be helpful to address this gap. Using a model of time-controllable ArchT expression, we demonstrated that the removal of ArchT resulted in the regression of SWDs (Fig. 8). These data indicate that the absence seizure phenotype is reversible in both humans and animal models and that a natural regression would be mediated by the disappearance of the disease’s cause. Most cases of human absence seizure are idiopathic, and therefore, it is possible that the transient (∼years) dysfunction in the cortico-thalamo-cortico pathway, including of the TRN, may cause absence seizure with natural regression in humans.

Another interesting point is the continuum of theta oscillation and SWDs. We observed that a high-power theta rhythm was the dominant rhythm in the cortical EEG of PV-ArchT mice compared with controls. We found a precedent expression of high-power theta rhythm after both virus-mediated (Fig. 5) and DOX regimen-mediated (Supplementary Fig. 2A-2C) ArchT induction. In the case of ArchT elimination by the DOX-off-to-on regimen, a reduction in theta rhythm power followed the disappearance of epileptiform discharges (Supplementary Fig. 2B). These data indicate that the high-power theta rhythm was dependent on the existence of ArchT in TRN, the lower level of ArchT expression was sufficient to induce high-power theta rhythm and a higher level of ArchT expression was required to induce epileptiform discharges.

TRN is believed to engage in the generation of delta waves^9,39^ and sleep spindles,^9,40,41^ but to our knowledge, there has been no report showing TRN dysfunction causes high-power theta waves. Despite the lack of association between TRN and theta rhythm, we may link them indirectly from the following evidence: TRN controls attention.^16^ Knockout of the TRN-selective expressing gene, *Ptchd1*, induces an attention deficit hyperactivity disorder (ADHD)-like phenotype. Knockout of the ADHD-associated (but ubiquitously expressed) gene, *Git1*, results in an ADHD phenotype in mice that exhibits enhanced theta rhythms.^42^ ADHD patients exhibit increased power of theta rhythm.^43,44^ Investigation of the potential link between high-power theta rhythm, TRN dysfunction and ADHD phenotype in TRN-related disease is warranted.

## Supplementary Material

fcac010_Supplementary_Data

## Abbreviations

AAV =: adeno-associated virus
AP =: anteroposterior
ArchT =: archaerhodopsin from Halorubrum strain TP009
CT =: corticothalamic
DOX =: doxycycline
DIG =: digoxigenin
DV =: dorsoventral
*GAERs* =: Genetic Absence Epilepsy Rat from Strasbourg
GFP =: green fluorescent protein
IHC =: immunohistochemistry
ISH =: *in situ* hybridization
LFP =: local field potential
ML =: mediolateral
P =: postnatal day
PBS =: phosphate-buffered saline
PV =: parvalbumin
SWD =: spike-and-wave discharge
TC =: thalamocortical
TRN =: thalamic reticular nucleus
tetO =: tetracycline operator site
tTA =: tetracycline transactivator
*WAG–Rij* =: Wistar Albino Glaxo Rijswijk
YC =: yellow cameleon

## References

[1] Fisher RS . The new classification of seizures by the International League Against Epilepsy 2017. Curr Neurol Neurosci Rep. 2017;17(6):48. 28425015 10.1007/s11910-017-0758-6

[2] Akman O , Demiralp T, Ates N, Onat FY. Electroencephalographic differences between WAG/Rij and GAERS rat models of absence epilepsy. Epilepsy Res. 2010;89(2-3):185–193. 20092980 10.1016/j.eplepsyres.2009.12.005

[3] Posner E . Absence seizures in children. BMJ Clin Evid. 2013;2013:0317. PMC3867171 24351614

[4] Crunelli V , Leresche N. Childhood absence epilepsy: Genes, channels, neurons and networks. Nat Rev Neurosci. 2002;3(5):371–382. 11988776 10.1038/nrn811

[5] Snead Iii OC . Basic mechanisms of generalized absence seizures. Ann Neurol. 1995;37(2):146–157. 7847856 10.1002/ana.410370204

[6] Pinault D , O’Brien TJ. Cellular and network mechanisms of genetically-determined absence seizures. Thalamus Relat Syst. 2005;3(3):181–203. 21909233 10.1017/S1472928807000209 PMC3168114

[7] Izhikevich EM , Edelman GM. Large-scale model of mammalian thalamocortical systems. Proc Natl Acad Sci USA. 2008;105(9):3593–3598. 18292226 10.1073/pnas.0712231105 PMC2265160

[8] Arcelli P , Frassoni C, Regondi MC, De Biasi S, Spreafico R. GABAergic neurons in mammalian thalamus: A marker of thalamic complexity? Brain Res Bull. 1997;42(1):27–37. 8978932 10.1016/s0361-9230(96)00107-4

[9] Steriade M , McCormick DA, Sejnowski TJ. Thalamocortical oscillations in the sleeping and aroused brain. Science. 1993;262(5134):679–685. 8235588 10.1126/science.8235588

[10] Krol A , Wimmer RD, Halassa MM, Feng G. Thalamic reticular dysfunction as a circuit endophenotype in neurodevelopmental disorders. Neuron. 2018;98(2):282–295. 29673480 10.1016/j.neuron.2018.03.021 PMC6886707

[11] Sasaki T , Beppu K, Tanaka KF, Fukazawa Y, Shigemoto R, Matsui K. Application of an optogenetic byway for perturbing neuronal activity via glial photostimulation. Proc Natl Acad Sci USA. 2012;109(50):20720–20725. 23185019 10.1073/pnas.1213458109 PMC3528589

[12] Tsunematsu T , Tabuchi S, Tanaka KF, Boyden ES, Tominaga M, Yamanaka A. Long-lasting silencing of orexin/hypocretin neurons using archaerhodopsin induces slow-wave sleep in mice. Behav Brain Res 2013;255:64–74. 23707248 10.1016/j.bbr.2013.05.021

[13] Kanemaru K , Sekiya H, Xu M, et al In vivo visualization of subtle, transient, and local activity of astrocytes using an ultrasensitive Ca^2+^ indicator. Cell Rep. 2014;8(1):311–318. 24981861 10.1016/j.celrep.2014.05.056

[14] Tanahira C , Fau HS, Watanabe K, et al Parvalbumin neurons in the forebrain as revealed by parvalbumin-Cre transgenic mice. Neurosci Res. 2009;63(3):213–223. 19167436 10.1016/j.neures.2008.12.007

[15] Tsutsui-Kimura I , Natsubori A, Mori M, et al Distinct roles of ventromedial versus ventrolateral striatal medium spiny neurons in reward-oriented behavior. Curr Biol. 2017;27(19):3042–3048.e4. 28966085 10.1016/j.cub.2017.08.061

[16] Wells MF , Wimmer RD, Schmitt LI, Feng G, Halassa MM. Thalamic reticular impairment underlies attention deficit in Ptchd1Y/^−^ mice. Nature. 2016;532(7597):58–63. 27007844 10.1038/nature17427 PMC4875756

[17] Tanaka KF , Samuels BA, Hen R. Serotonin receptor expression along the dorsal-ventral axis of mouse hippocampus. Philos Trans R Soc Lond B Biol Sci. 2012;367(1601):2395–2401. 22826340 10.1098/rstb.2012.0038 PMC3405677

[18] Jarre G , Guillemain I, Deransart C, Depaulis A. Chapter 32—Genetic models of absence epilepsy in rats and mice. In: Pitkänen A Buckmaster PS Galanopoulou AS, Moshé SL, eds. Models of seizures and epilepsy, 2nd edn. Academic Press; 2017:455–471.

[19] Brigo F , Igwe SC. Ethosuximide, sodium valproate or lamotrigine for absence seizures in children and adolescents. Cochrane Database Syst Rev. 2017;2:CD003032. 28195639 10.1002/14651858.CD003032.pub3 PMC6464603

[20] Cain SM , Garcia E, Waheed Z, Jones KL, Bushell TJ, Snutch TP. GABAB receptors suppress burst-firing in reticular thalamic neurons. Channels (Austin). 2017;11(6):574–586. 28742985 10.1080/19336950.2017.1358836 PMC5786188

[21] Aizawa M , Ito Y, Fukuda H. Roles of gamma aminobutyric acid and gamma-hydroxybutyric acid receptors in hippocampal long-term potentiation and pathogenesis of absence seizures. Biol Pharm Bull. 1997;20(10):1066–1070. 9353566 10.1248/bpb.20.1066

[22] Chow BY , Han X, Dobry AS, et al High-performance genetically targetable optical neural silencing by light-driven proton pumps. Nature. 2010;463(7277):98–102. 20054397 10.1038/nature08652 PMC2939492

[23] Yamazaki Y , Abe Y, Shibata S, et al Region- and cell type-specific facilitation of synaptic function at destination synapses induced by oligodendrocyte depolarization. J Neurosci. 2019;39(21):4036. 30862665 10.1523/JNEUROSCI.1619-18.2019 PMC6529859

[24] Contreras D , Curro Dossi R, Steriade M. Bursting and tonic discharges in two classes of reticular thalamic neurons. J Neurophysiol. 1992;68(3):973–977. 1432063 10.1152/jn.1992.68.3.973

[25] Jahnsen H , Llinás R. Electrophysiological properties of guinea-pig thalamic neurones: An in vitro study. J Physiol. 1984;349:205–226. 6737292 10.1113/jphysiol.1984.sp015153 PMC1199334

[26] Clemente-Perez A , Makinson SR, Higashikubo B, et al Distinct thalamic reticular cell types differentially modulate normal and pathological cortical rhythms. Cell Rep. 2017;19(10):2130–2142. 28591583 10.1016/j.celrep.2017.05.044 PMC5557038

[27] Nestler EJ , Hyman SE. Animal models of neuropsychiatric disorders. Nat Neurosci. 2010;13(10):1161–1169. 20877280 10.1038/nn.2647 PMC3750731

[28] Kandratavicius L , Balista PA, Lopes-Aguiar C, et al Animal models of epilepsy: Use and limitations. Neuropsychiatr Dis Treat. 2014;10:1693–1705. 25228809 10.2147/NDT.S50371 PMC4164293

[29] Puranam RS , McNamara JO. Seizure disorders in mutant mice: Relevance to human epilepsies. Curr Opin Neurobiol. 1999;9(3):281–287. 10395577 10.1016/s0959-4388(99)80041-5

[30] Noebels JL , Fariello RG, Jobe PC, Lasley SM, Marescaux C. Genetic models of generalized epilepsy. In: Pedley JE and Pedley TA, eds. Epilepsy: A comprehensive textbook. Lippincott-Raven; 1997:457–465.

[31] Cox GA , Lutz CM, Yang C-L, et al Sodium/hydrogen exchanger gene defect in slow-wave epilepsy mutant mice. Cell. 1997;91(1):139–148. 9335342 10.1016/s0092-8674(01)80016-7

[32] Sherman SM . Tonic and burst firing: Dual modes of thalamocortical relay. Trends Neurosci. 2001;24(2):122–126. 11164943 10.1016/s0166-2236(00)01714-8

[33] Cain SM , Snutch TP. T-type calcium channels in burst-firing, network synchrony, and epilepsy. Biochim Biophys Acta. 2013;1828(7):1572–1578. 22885138 10.1016/j.bbamem.2012.07.028

[34] Talley EM , Cribbs LL, Lee J-H, Daud A, Perez-Reyes E, Bayliss DA. Differential distribution of three members of a gene family encoding low voltage-activated (T-type) calcium channels. J Neurosci. 1999;19(6):1895–1911. 10066243 10.1523/JNEUROSCI.19-06-01895.1999 PMC6782581

[35] Lee SE , Lee J, Latchoumane C, et al Rebound burst firing in the reticular thalamus is not essential for pharmacological absence seizures in mice. Proc Natl Acad Sci USA. 2014;111(32):11828–11833. 25071191 10.1073/pnas.1408609111 PMC4136605

[36] Chen Y , Lu J, Pan H, et al Association between genetic variation of CACNA1H and childhood absence epilepsy. Ann Neurol. 2003;54(2):239–243. 12891677 10.1002/ana.10607

[37] Astori S , Wimmer RD, Prosser HM, et al The CaV3.3 calcium channel is the major sleep spindle pacemaker in thalamus. Proc Natl Acad Sci USA. 2011;108(33):13823–13828. 21808016 10.1073/pnas.1105115108 PMC3158184

[38] Cheong E , Shin HS. T-type Ca2^+^ channels in normal and abnormal brain functions. Physiol Rev. 2013;93(3):961–992. 23899559 10.1152/physrev.00010.2012

[39] Lewis LD , Voigts J, Flores FJ, et al Thalamic reticular nucleus induces fast and local modulation of arousal state. Elife. 2015;4:e08760. 26460547 10.7554/eLife.08760 PMC4686423

[40] Halassa MM , Siegle JH, Ritt JT, Ting JT, Feng G, Moore CI. Selective optical drive of thalamic reticular nucleus generates thalamic bursts and cortical spindles. Nat Neurosci. 2011;14(9):1118–1120. 21785436 10.1038/nn.2880 PMC4169194

[41] Thankachan S , Katsuki F, McKenna JT, et al Thalamic reticular nucleus parvalbumin neurons regulate sleep spindles and electrophysiological aspects of schizophrenia in mice. Sci Rep. 2019;9(1):3607. 30837664 10.1038/s41598-019-40398-9 PMC6401113

[42] Won H , Mah W, Kim E, et al GIT1 is associated with ADHD in humans and ADHD-like behaviors in mice. Nat Med. 2011;17(5):566–572. 21499268 10.1038/nm.2330

[43] Barry RJ , Clarke AR, Johnstone SJ. A review of electrophysiology in attention-deficit/hyperactivity disorder: I. Qualitative and quantitative electroencephalography. Clin Neurophysiol. 2003;114(2):171–183. 12559224 10.1016/s1388-2457(02)00362-0

[44] Mann CA , Lubar JF, Zimmerman AW, Miller CA, Muenchen RA. Quantitative analysis of EEG in boys with attention-deficit-hyperactivity disorder: Controlled study with clinical implications. Pediatr Neurol. 1992;8(1):30–36. 1558573 10.1016/0887-8994(92)90049-5

