# Supplementary figures and images for: Dysfunction of parvalbumin-expressing cells in the thalamic reticular nucleus induces cortical spike-and-wave discharges and an unconscious state
Source: Brain Commun. 2022 Jan 28;4(2):fcac010. doi: 10.1093/braincomms/fcac010 (PMC8887905; doi:10.1093/braincomms/fcac010)

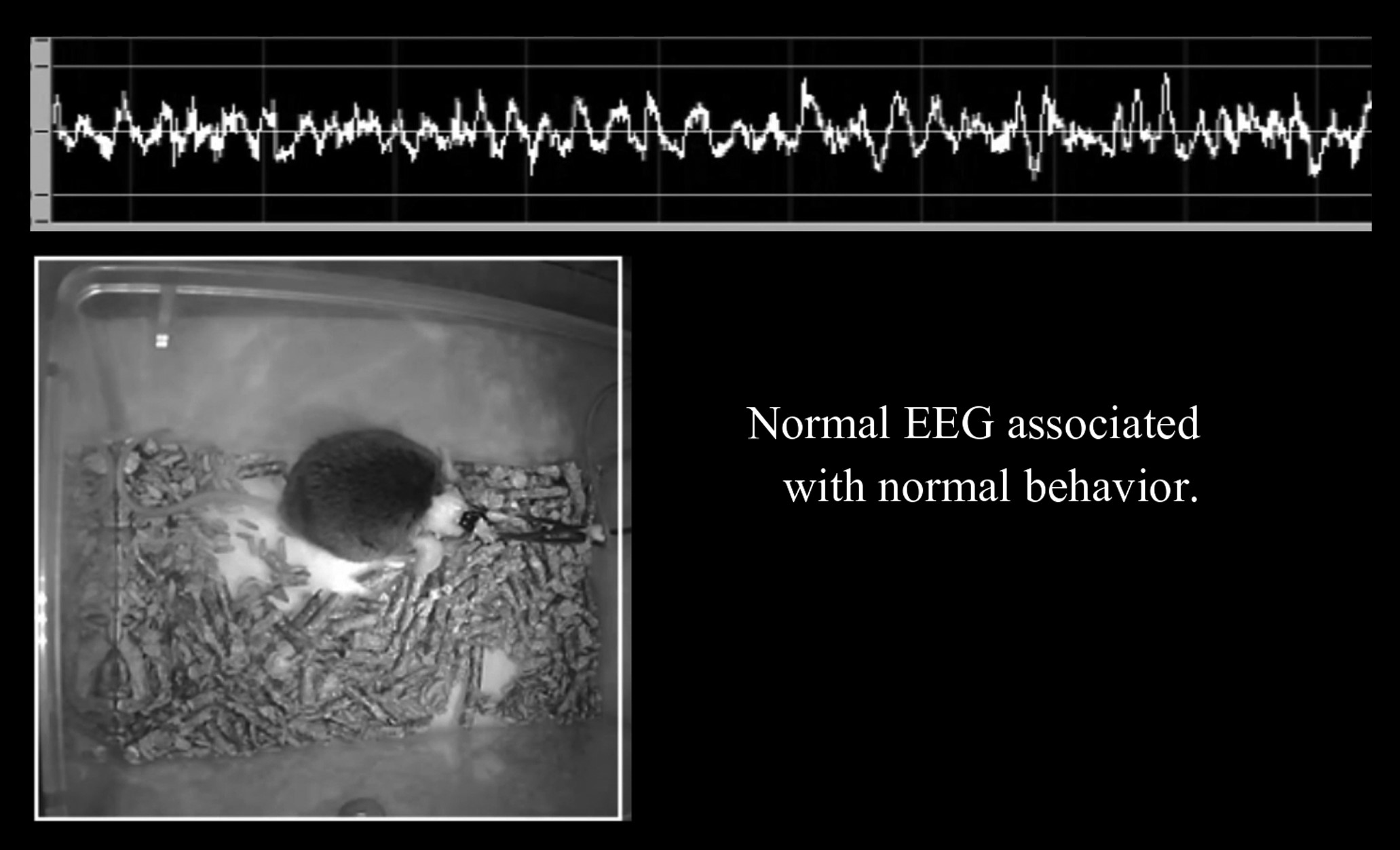

Supplement: Supplementary file 1 [file fcac010v1.jpeg]
