# Supplementary material for: Dysfunction of parvalbumin-expressing cells in the thalamic reticular nucleus induces cortical spike-and-wave discharges and an unconscious state
Source: Brain Commun. 2022 Jan 28;4(2):fcac010. doi: 10.1093/braincomms/fcac010 (PMC8887905; doi:10.1093/braincomms/fcac010)
Supplement: fcac010_Supplementary_Data [file fcac010_supplementary_data.docx]

**Supplementary materials**

**Supplementary Figure 1**


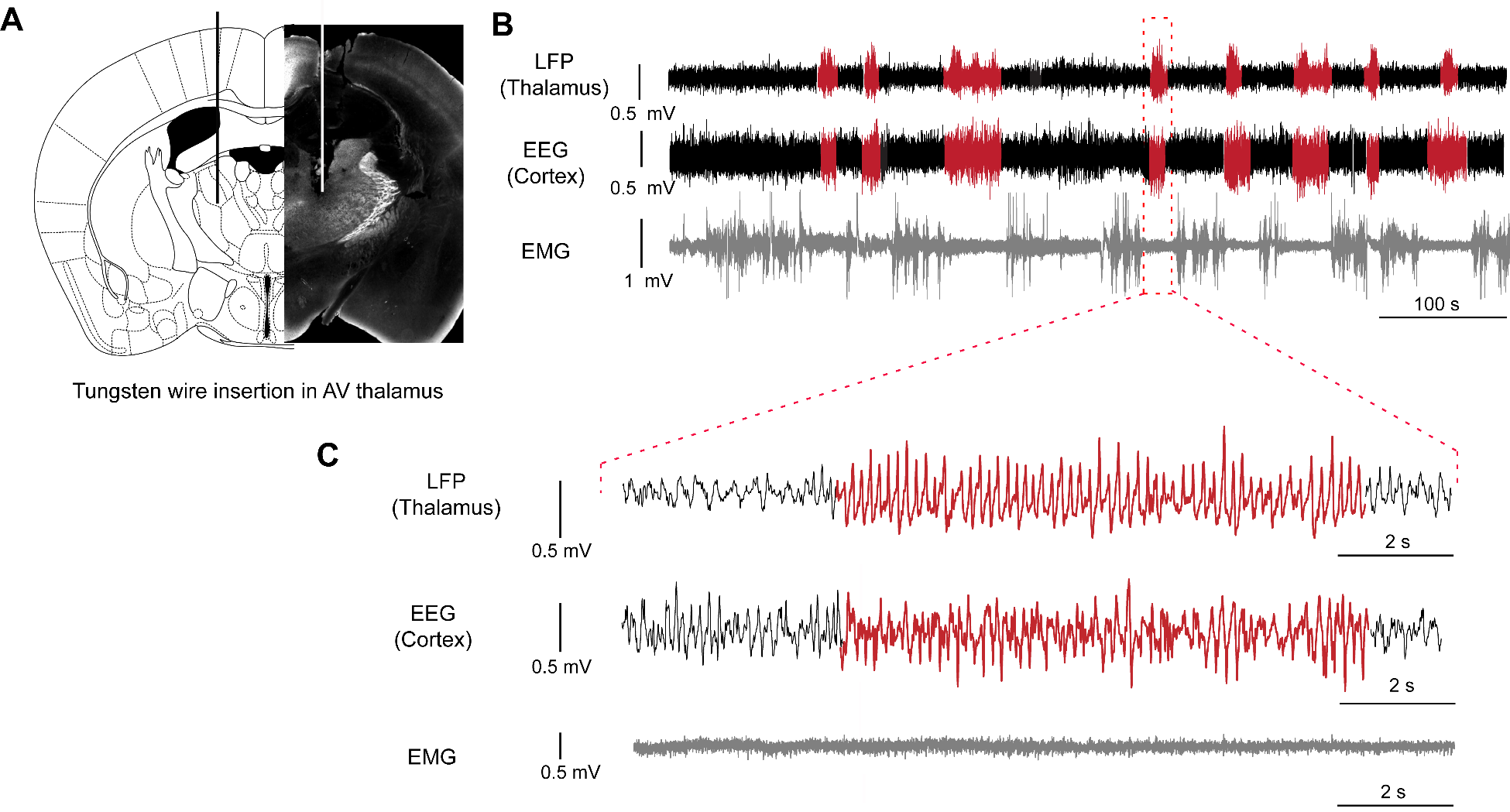


**Supplementary Figure 1. Thalamic hyperactivity coincided with cortical spike-and-wave discharge (SWDs) in PV-ArchT mice.**

(**A**) The location of a tungsten electrode in the anteroventral thalamus (AV) (**B**) The top trace shows thalamic LFPs with hyperactive events (red traces) that coincided with cortical SWDs (87 ± 1.4%, n = 6 records from 3 mice). The middle and the bottom traces show EEG and EMG, respectively. (**C**) Expanded traces of thalamic local field potential (LFP), cortical EEG, and EMG from the red rectangle in panel A. Red traces show the thalamic hyperactivity and cortical SWDs, respectively.

**Supplementary Figure 2**


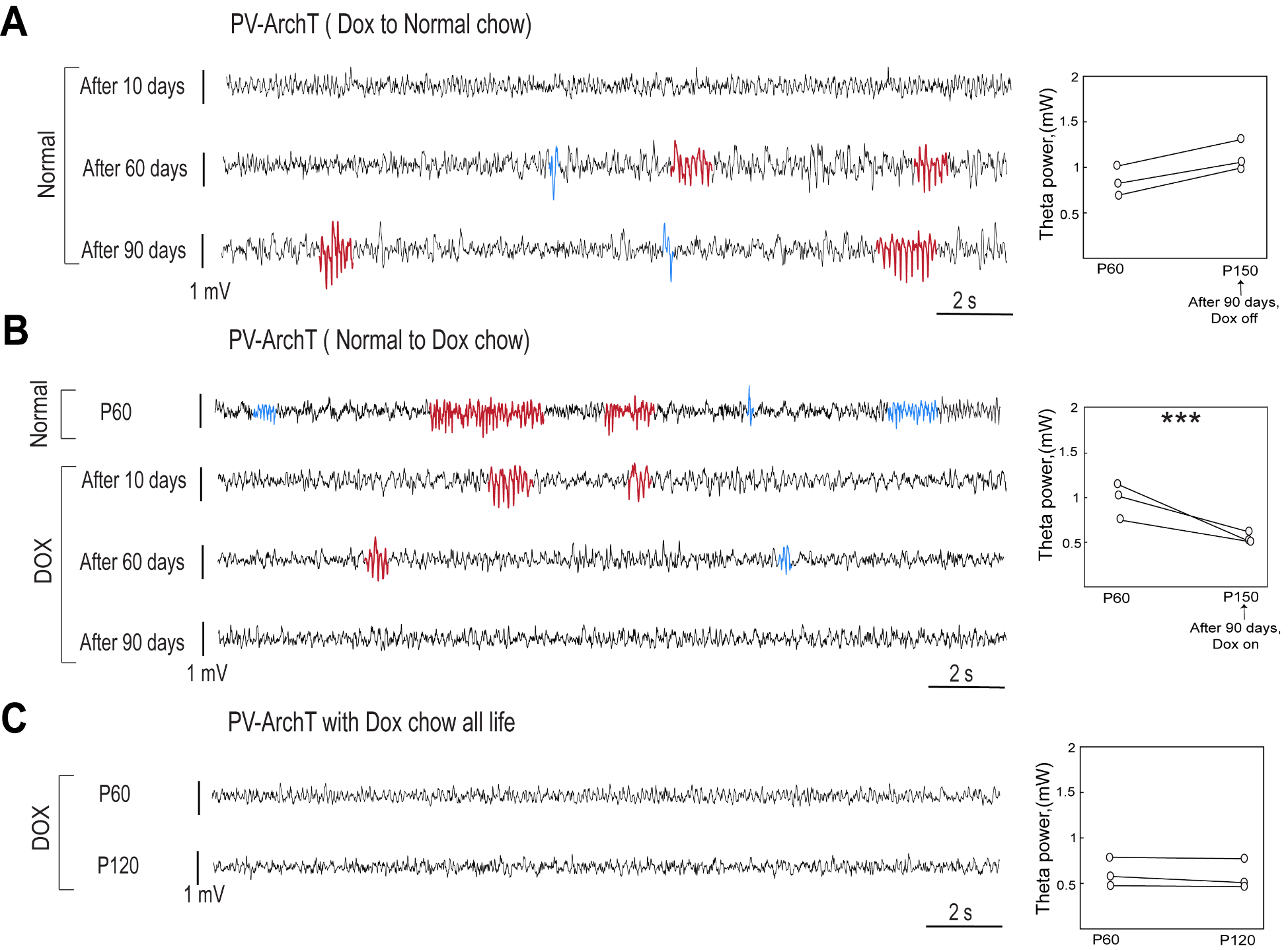


**Supplementary Figure 2. Cortical EEG changes were dose and time-dependently induced by ArchT induction.** Representative cortical EEG traces (left) are shown. SWDs and other types of epileptiform discharges are marked by red and blue, respectively. Line plots show basal theta power changes with age. **(A)** Pattern D in Figure 8. There was a trend toward increased theta power after normal chow consumption (paired *t* test, *P* = 0.097, n = 3 mice). **(B)** Pattern B in Figure 8. High-power theta declined after DOX administration (paired *t* test, *P* = 0.033, n = 3 mice). **(C)** Pattern C in Figure 8. No change in theta power (paired *t* test, *P* = 0.438, n = 3 mice).

**Supplementary Table 1**

| **Table S1: Membrane properties of PV neurons in PV-ArchT.** | | | | | | |
| --- | --- | --- | --- | --- | --- | --- |
|  | Membrane time constant (ms) | Membrane capacitance (pF) | AP threshold (mV) | Series resistance (Mohm) | # Cells | # Mice |
| PV-ArchT | 2.74 ± 0.13 | 17.8 ± 1.28 | -37.8 ± 0.63 | 15.2 ± 1.12 | 20 | 3 |
| Control | 3.23 ± 0.22 | 20.4 ± 1.01 | -38.6 ± 2.61 | 13.5 ± 2.09 | 10 | 3 |
| t-test | -1.58 | -1.57 | 0.28 | 0.60 | - | - |
| P-value | 0.22 | 0.19 | 0.81 | 0.53 | - | - |

**Table S1: Important metrics of intrinsic excitability of PV neurons in PV-ArchT and control, related to** **Figure 6**. None of the measures showed any significant difference. Data represented as mean ± SEM.
